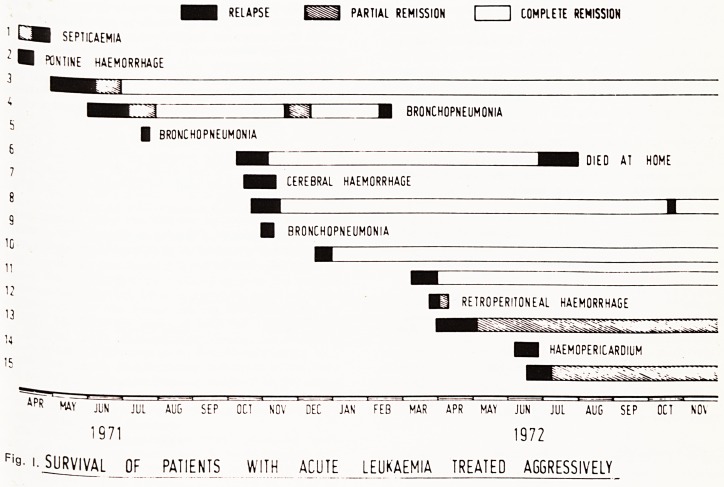# Adult Acute Leukaemia—A Suitable Case for Treatment?

**Published:** 1973-01

**Authors:** T. J. Hamblin, J. Verrier Jones

**Affiliations:** University Department of Medicine, Southmead Hospital, Bristol; University Department of Medicine, Southmead Hospital, Bristol


### Adult Acute Leukæmia—a suitable case for treatment?

**T. J. Hamblin* and J. Verrier Jones**
University Department of Medicine, Southmead Hospital, Bristol

### INTRODUCTION

Many physicians have, in the past, doubted the value of aggressive treatment for acute leukaemia in adults. This view has had much justification. Between 1948 (Tivey, 1954) and 1966 (M.R.C. Working Party, 1966) the average survival had remained constant at around 70 days, despite the fact that antibiotics and blood transfusion had been supplemented by cytotoxic drugs capable of inducing remission in over 80% of children with acute leukaemia (Acute Leukaemia Group B, 1965). By 1965 Burchenal was already talking of the prospect of cure in childhood acute leukaemia and cited 53 patients who had survived longer than five years (Burchenal et al. 1965). However, he could find only six adults in whom there was even a possibility of cure.

Skipper's experiments on the transplanted murine leukaemia (Skipper et al., 1964) revealed an important difference between normal and abnormal cells that could be used in treatment. Normal cells grow more rapidly, and therefore, if a large number of normal and abnormal cells are killed, the normal cells should recover first, allowing a further course of treatment to be given. Successive courses of treatment would reduce the numbers of leukaemic cells to levels that could be handled by the body's own immune mechanisms.

Experimental treatment schedules based on this principle were quick to appear, and have been successful in prolonging life in patients treated at research centres (Crowther et al., 1970).

We set out to examine whether such heroic treatment schedules were applicable in the context of a district general hospital.

### PATIENTS AND METHODS

Over an eighteen month period 15 patients were admitted suffering from acute leukaemia. There were five males and ten females and the age range was 22 to 82, with a mean age of 50.

Details are given in Table I.

Diagnosis was established by bone marrow examination, using special cytochemical stains as required, in consultation with Dr. F. J. W. Lewis and Dr. I. Fraser. No patient was excluded from treatment on grounds of age or clinical condition. Treatment schedules were chosen in consultation with the haematologist, and with Dr. J. S. Malpas of St. Bartholomew's Hospital, and were tailored to suit individual patients.

Initial investigations included blood urea and plasma electrolytes, urine analysis, electrocardiography, chest X-ray, liver function tests, serum calcium, phosphate and uric acid. Progress was followed by blood counts on alternate days, and bone marrow examination prior to each course of treatment. Haematological results were charted on semi-logarithmic graph paper for ease of assessment.

All patients had a full bacteriological survey in the form of swabs from nose, throat, ears, umbilicus and perineum, and cultures of blood and urine.

**Cytotoxic drugs used** (Table II). Patients with acute myeloblastic leukaemia and erythro-leukaemia were treated with cytosine arabinoside 2 mg/kg by intravenous injection daily for five days, with daunorubicin 1.5 mg/kg by fast intravenous infusion on day 1. This was repeated every 10 days until blast cells disappeared from the peripheral blood and bone marrow. After remission, maintenance treatment was given every 6 weeks, consisting of cytosine arabinoside and daunorubicin as above, alternating with cytosine arabinoside or 6-thioguanine 2 mg/kg orally for 5 days. In the event of relapse, methotrexate, 6-mercaptopurine or cyclophosphamide were added to these schedules.

One patient with acute lymphoblastic leukaemia was treated by the technique described by Aur et al. (1971).

Marrow depression was anticipated and treated in the following manner.

**Anaemia** was treated when symptomatic by the transfusion of packed red cells.

**Thrombocytopenia** was treated initially only when accompanied by signs of a haemorrhagic diathesis, such as bruising, purpura or frank bleeding. At a later stage, when platelets for transfusion became more easily available, we treated when the platelet count fell below 20,000/$\mu$l. Platelet concentrates from 4-6 donors were injected intravenously daily as necessary.

**Neutropenia** was treated expectantly. When the neutrophil polymorph count fell below 500/$\mu$l we instituted reversed barrier nursing in cubicles off the general ward. Infections were treated with appropriate antibiotic after identification of the organism, and after consultation with the microbiologist, Dr. D. S. Reeves. Occasionally it was necessary to start treatment before an organism had been identified, and a suitable combination of antibiotics was used, again on the advice of Dr. Reeves. We did not use prophylactic antibiotics, nor did we attempt to sterilise the patient's bowel or his food.

### RESULTS

Of the fifteen patients, six achieved a complete remission (normal blood count and normal bone marrow with a return to normal life). Two achieved partial

*Present address: Department of Pathology, Poole General Hospital, Poole, Dorset, BH15 2JB.

TABLE I. DETAILS OF PATIENTS TREATED

| No. | Age | Sex | Diagnosis | Platelets 1μ1 | Blasts 1μ1 | Remission Inducing Drugs | No. of Courses | | Weeks | Maintenance Drugs |
|---|---|---|---|---|---|---|---|---|---|---|
| 1 | 80 | M | AML | 80,000 | 20 | ARA-C | 1 | ED | — | — |
| 2 | 82 | F | AML | 10,000 | 14,000 | ARA-C | 1 | ED | — | — |
| 3 | 28 | M | EL | 120,000 | 700 | ARA-C, DR | 4 | CR | 69 | DR, 6TG, ARA-C |
| 4 | 59 | F | AML | 25,000 | 25,000 | ARA-C, DR | 4 | CR | 30 | DR, 6TG, ARA-C |
| 5 | 38 | M | AML | 28,000 | 14,000 | ARA-C, DR | 1 | ED | | |
| 6 | 78 | F | AML | 10,000 | 100,000 | ARA-C, DR | 3 | CR | 32 | 6TG, ARA-C |
| 7 | 37 | M | AML | 20,000 | 800 | ARA-C, DR | 2 | TD | — | — |
| 8 | 48 | F | AML | 68,000 | 18,000 | ARA-C, DR | 2 | CR | 47 | DR, 6TG, ARA-C |
| 9 | 65 | M | SCL | 10,000 | 90 | ARA-C, DR, PRED | 1 | ED | — | — |
| 10 | 22 | F | ALL | 12,000 | 10,000 | VCR, PRED | 4 | CR | 41 | CYC, 6MP, VCR, PRED MTX, DXT |
| 11 | 34 | F | SCL | 28,000 | 2,500 | VCR PRED DR ARA-C | 2 | CR | 30 | VCR, PRED DR, ARA-C |
| 12 | 28 | F | AML | 20,000 | 1,800 | ARA-C, DR | 1 | TD | — | — |
| 13 | 62 | F | EL | 78,000 | 50 | ARA-C, DR | 2 | PR | 23 | VCR, PRED DR, ARA-C |
| 14 | 50 | M | AMML | 10,000 | 5,000 | ARA-C, DR | 2 | TD | — | — |
| 15 | 66 | F | AMML | 20,000 | 1,600 | ARA-C, DR | 3 | PR | 14 | ARA-C, DR |

AML—Acute Myeloblastic Leukaemia, EL—Erythroleukaemia, SCL—Stem Cell Leukaemia, AMML—Acute Myelomonocytic Leukaemia, ALL—Acute Lymphoblastic Leukaemia.
ARA-C—Cystosine Arabinoside, DR—Daunorubicin, VCR—Vincristine, 6TG—6 Thioguanine, MTX—Methotrexate, DXT—Radiotherapy to skull.
ED—early death, TD—treatment death, CR—complete remission, PR—partial remission.

remissions, with return to normal life, and disappearance of blasts from peripheral blood and bone marrow, but accompanied by a degree of marrow hypoplasia, reflected in neutropenia and thrombocytopenia in the peripheral blood.

There were four deaths before the completion of the first course of treatment. In three cases death resulted from overwhelming infection that had been present at diagnosis, and in the fourth a pontine haemorrhage was associated with a pre-existing thrombocytopenia. There were three treatment deaths during the phase of marrow aplasia, all from haemorrhage associated with thrombocytopenia. These results are summarised in Table I.

early deaths all required blood transfusion. A total of 165 units of packed red cells were transfused, an average of 11 units per patient. One patient developed a red cell isoantibody following blood transfusion. All patients achieving a remission were able to maintain a normal haemoglobin.

**Thrombocytopenia.** The platelet count at presentation is given in Table I. Those who were not already thrombocytopenic rapidly became so on treatment. A total of 200 units of platelet concentrate were given, the majority prophylactically because of a low platelet count.

Two patients became refractory to platelets and died of haemorrhage, one intracerebral, and the other retro-

Fig. I. SURVIVAL OF PATIENTS WITH ACUTE LEUKAEMIA TREATED AGGRESSIVELY

**Length of remission.** This is shown graphically in Figure 1. Four of the patients have relapsed after 19, 32, 47 and 28 weeks. In one a second remission lasting for eleven weeks was obtained when a further relapse occurred; attempts at a third remission were unsuccessful. Four patients are in remission after 27, 18, 34 and 73 weeks.

**Complications of treatment.** Bone marrow hypoplasia is a necessary consequence of successful treatment, and occurred in all patients surviving the first course of drugs. Severe hypoplasia lasted between one and six weeks in those achieving a remission, with an average of two weeks.

**Anaemia.** All patients were anaemic at presentation and became more so on treatment. Apart from two peritoneal. A further patient died of haemorrhage into the pericardial cavity despite the fact that donor platelets had raised his count to 34,000/$\mu$l. There was one other case of severe haemorrhage, this time into middle and inner ear, which was arrested after platelet transfusion. The patient survived for nearly nine months after the episode, and despite being totally deaf she fitted well as an aged grandmother into a household which luckily made little use of verbal communication.

**Neutropenia.** This was also a regular feature in those who survived the first course of treatment, and all of these patients were barrier nursed at some stage of their treatment. Fifteen patients spent 42 patient weeks in isolaion.

All the patients surviving the first course of treatment suffered infective complications, though most of them were minor. The mouth was the most common site. There were six episodes of gingival or peridontal infection, two of tonsilitis, two of labial herpes simplex, two of oral candidiasis, and one of parotitis.

Two of the infective episodes occurred after medical interference; an infected infusion site led to a staphylococcal septicaemia, and a staphylococcal abscess occurred at the site of an intramuscular injection.

There were two cases of bronchopneumonia, and a middle ear infection. All of the other bacterial infections began in the skin. A staphylococcal septicaemia started life as a pustule on a finger, and a patient presenting with perineal ulceration developed perineal cellulitis.

There were in addition two episodes of presumed viral infection which were asymptomatic and self-resolving. The patients presented with neutropenia, thrombocytopenia and an atypical mononucleosis while safely in remission. Bone marrow examination showed an infiltration with atypical mononuclear cells, but no sign of relapse. Tests for infectious mononucleosis, toxoplasmosis and cytomegalovirus all proved negative.

There were no deaths from infection in patients made neutropenic by the treatment.

**Depression.** None of the patients enjoyed being isolated in cubicles and four complained of depression. In addition, one patient suffered a severe depressive psychosis which required psychiatric help.

**Neurological.** None of the patients receiving vincristine developed a peripheral neuropathy, but the patient who had cranio-spinal irradiation developed a radiculitis which interfered with walking. This was slow to resolve but the patient (10) is now leading a normal life.

**Other drug reactions.** One patient had an urticarial reaction to flucloxacillin, and another suffered acute renal failure after being treated with the combination of frusemide, gentamicin and cephaloridine. Both of these patients made a complete recovery. Most patients receiving cytosine arabinoside complained of transient nausea. One patient had T wave inversion in her ECG after daunorubicin, but this was asymptomatic.

**Quality of life in remission.** Those patients achieving a remission were able to return to their normal occupation if they desired. Ideally the only medical demands on their time were weekly venepunctures, and a weekend admission every six weeks for bone marrow examination, and daunorubicin infusion. Cytosine arabinoside may be given subcutaneously, and patients may be trained to inject themselves as diabetics do.

Some patients elected not to return to their old routine but to enjoy an extended holiday. One patient devoted her last six months to raising money for cancer research. On the other hand, one patient has been running his own business successfully for over a year since his illness.

All the patients felt subjectively well. Indeed, some of them experienced difficulty in getting their families and friends to reconcile their apparent well-being with their sinister diagnosis.

TABLE II DETAILS OF DRUGS EFFECTIVE IN ACUTE LEUKAEMIA

| Drug | Source | Action | Target | Side Effects | Route |
|---|---|---|---|---|---|
| Vincristine | Alkaloid of periwinkle | Inhibits spindle formation | Lymphoid | Alopecia<br>Peripheral neuropathy | i/v |
| Prednisolone | Adreno-cortical steroid | Prevents entry into S phase | Lymphoid | Cushing's<br>Psychosis<br>Immuno-suppression | oral |
| Cyclophosphamide | Synthetic alkylating agent | Alkylates DNA | Lymphoid | Alopecia<br>Cystitis | i/v<br>oral |
| L—Asparaginase | Enzyme of *Esch. coli* | Renders asparagine unavailable | Lymphoid | Hyper-sensitivity<br>Nausea | i/v |
| Methotrexate | Synthetic folate acid antagonist | Inhibits thymidilate synthesis | Lymphoid and Myeloid | Oral ulcers<br>Liver damage | i/v<br>oral |
| 6—Mercaptopurine and 6—Thioguanine | Synthetic purine antagonists | Inhibits DNA synthesis | Lymphoid and Myeloid | Gastro-intestinal | oral |
| Daunorubicin | Antibiotic from *Streptomyces coeruleorubidus* | Complexes with DNA | Myeloid | Cardiac | i/v |
| Cytosine Arabinoside | Synthetic pyrimidine antagonist | Inhibits DNA synthesis | Myeloid | Gastro-intestinal | i/v |

### DISCUSSION

A few specialised centres have reported promising results from the aggressive treatment of adult acute leukaemia (Crowther et al., 1970; Carey, 1970; M.R.C. Working Party, 1971). The treatment itself is hazardous, and it is necessary to assess whether it is appropriate to introduce it into a district general hospital. A study of children with acute lymphoblastic leukaemia shows that results are better when the patients are treated in a specialised centre.

Published series showing good results are often small and selected. There is usually an age limit above which treatment is not offered, and in some series the more malignant variants such as erythroleukaemia and monocytic leukaemia are excluded. Many series comprise mainly referred cases, and consequently exclude those patients whose clinical condition is so poor on diagnosis as to preclude referral.

Our series, though small, is unselected. Nevertheless, our remission rate of 55% compares favourably with the best yet reported (Crowther et al. 1970).

However, it is not enough to prolong life. The life must be worth living. We have therefore been careful to assess the morbidity involved in this kind of treatment, and also the quality of life achieved when a remission is obtained.

There is no doubt that most patients undergo discomfort during the phase of marrow hypoplasia. A few will die during this period. It is significant that in our series more died of their disease before the treatment was effective.

The period of hypoplasia lasts on average two weeks, but some patients may have to endure it for considerably longer. We anticipate this period and take steps to reduce the discomfort by discussing with the patient the need for isolation, by attempting to maintain sensible contact with the patient during the period, and by constant vigilance for signs of infection. Undoubtedly the greatest cause of discomfort during this period is oral ulceration, which has a disturbingly high incidence. The quality of life during remission is excellent and many patients are in better health than for some time before their illness. All patients achieving a remission thought the rigours of the treatment worthwhile in view of the result obtained.

We are therefore convinced that aggressive treatment of adult acute leukaemia is both feasible and practicable in a district general hospital. We have been able to offer fifteen patients, all otherwise close to death, over five years of normal life between them, and feel that this result justifies this therapeutic approach.

### SUMMARY

We have treated fifteen adults with acute leukaemia using an aggressive regime. Eight of the patients achieved remission of their disease. While there is a certain mortality and morbidity associated with the treatment, those achieving remission have been able to live normal lives for periods of up to eighteen months.

### ACKNOWLEDGEMENTS

We are grateful to our colleagues for referring cases, and to Dr. F. J. W. Lewis and Dr. Ian Fraser for haematological advice, to the South West Regional Blood Transfusion Centre for their help, and to Dr. D. S. Reeves for bacteriological advice.

### REFERENCES

1. Acute Leukaemia Group B. (1965), New treatment schedule with improved survival in childhood leukaemia. Journal of the American Medical Association, **194**, 75-81.

2. Burchenal, J. H. and Murphy, M. L. (1965), Long term survivors in acute leukaemia. Cancer Research, **25**, 1491-1494.

3. Carey, R. W. (1970). Comparative study of cytosine arabinoside therapy alone and combined with thioguanine, mercaptopurine or daunomycin in acute myelocytic leukaemia. Proceedings of the American Association for Cancer Research, **11**, 15.

4. Crowther, D., Bateman, C. J. T., Vartan, C. P., Whitehouse, J. M. A., Malpas, J. S. Hamilton Fairley, G. and Bodley Scott, R. (1970). Combination chemotherapy using L-Asparaginase, Daunorubicin and Cytosine Arabinoside in adults with acute myelogenous leukaemia. British Medical Journal, **iv**, 513-517.

5. Report of M.R.C. Working Party (1966), Treatment of acute leukaemia in adults : comparison of steroid and mercaptopurine therapy, alone and in conjunction. British Medical Journal, **i**, 1383-1389.

6. Report of M.R.C. Working Party (1971), Treatment of acute lymphoblastic leukaemia. British Medical Journal, **iv**, 189-194.

7. Skipper, H. E., Schabel, F. M. and Wilcox, W. S. (1964). Experimental evaluation of potential anticancer agents. XIII. On the criteria and kinetics associated with "curability" of experimental leukaemia. Cancer Chemotherapy Reports, **35**, 1-111.

8. Tivey, H. (1954), The natural history of untreated acute leukaemia. Annals New York Academy of Science, **60**, 322-358.

## Figures and Tables

**Fig. 1. f1:**